# Neutron Total Scattering Experiments at High Pressure: Measuring Crystalline Structural Heterogeneity using the SNAP Instrument at the Spallation Neutron Source

**DOI:** 10.1063/4.0000895

**Published:** 2025-10-27

**Authors:** John Hirtz, Cale Overstreet, Eric C O'Quinn, Maik K Lang, A. M. dos Santos, Matthew Tucker

**Affiliations:** 1 University of Tennessee, Knoxville, TN, USA; 2 Oak Ridge National Laboratory, Oak Ridge, TN, USA

## Abstract

Exposure of materials to extreme pressure induces complex structural distortions, disorder, and phase transformations which effect their electronic, magnetic, and mechanical properties. These structural deviations, however, can be challenging to characterize with Bragg scattering alone. Neutron total scattering, with an increased sensitivity to low-*Z* elements, is a powerful tool for analyzing these changes by revealing local atomic configurations. Despite this, total scattering experiments at extreme pressure have remained rare in literature due to the increased experimental complexity compared with total scattering experiments at ambient conditions. In the experiments that have been performed, *Q-*ranges are limited due to the more complex neutron attenuation caused by the changing instrument geometry and gasket deformation. We have investigated the potential for performing similar experiments at the SNAP instrument at the Spallation Neutron Source at Oak Ridge National Laboratory. SNAP is an ideal instrument for total scattering as it boasts the most intense neutron flux among beamlines specialized for high pressure work. Further, spallation sources are ideal for total scattering experiments as they provide higher energy neutrons than available at reactor sources which extends the potential *Q*-range of the measurement. We have used the SNAP instrument to characterize Y_2_Zr_2_O_7_, which is a disordered fluorite-structured oxide with a distinct local Weberite-type (characteristic of A_3_BO_7_ oxides) phase that is not detectable through long range structural techniques. The quality of the captured total scattering data at ambient conditions and at high pressure is discussed and compared to data collected on NOMAD, a dedicated neutron total scattering instrument at the same facility.

